# Anisotropic dual-plasmonic hetero-nanostructures with tunable plasmonic coupling effects†

**DOI:** 10.1039/d2na00126h

**Published:** 2022-04-12

**Authors:** Mariia Ivanchenko, Hao Jing

**Affiliations:** Department of Chemistry and Biochemistry, George Mason University Fairfax VA 22030 USA hjing2@gmu.edu

## Abstract

The influence of plasmonic coupling effects between different components in Au NRs@Cu_2−*x*_Se nanostructures on their characteristics was studied. To this aim, anisotropic Au@Cu_2−*x*_Se hetero-nanostructures with well-controlled design and optical properties were obtained. The LSPR bands of gold and copper selenide are superpositioned in the NIR region, resulting in superior photocatalytic properties of the nanostructures.

## Abstract 2

The influence of plasmonic coupling effects between different components in Au NRs@Cu_2−*x*_Se nanostructures on their characteristics was studied.

## Graphical abstract

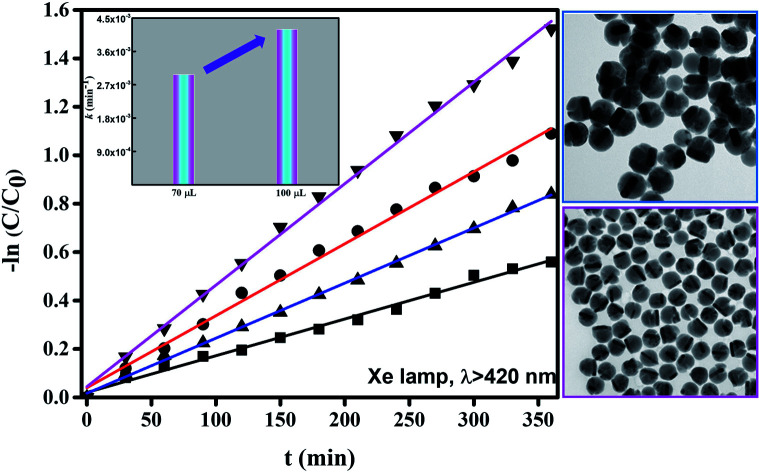

Nowadays, hetero-nanostructures with various compositions and morphologies have received scientific attention due to generated synergistic effects and promising multifunctional physicochemical properties.^1–6^ Specifically, noble metal–semiconductor heterostructures harnessing the phenomenon of localized surface plasmon resonance are utilized for a broad range of applications including photocatalysis,^7,8^ photovoltaics,^9,10^ water splitting,^11,12^ optics,^13^ and biomedicine.^14–17^ In addition to noble metals, which support LSPRs in the visible spectral range and are traditionally used in plasmonics,^18^ vacancy-doped copper chalcogenides are actively investigated for prospective utilization in multiple fields.^19,20^ Unlike plasmons formed by the collective oscillation of free electrons in metals, plasmons in such semiconductors arise due to the presence of free holes.^21^ Considering the abundant hole carriers in copper-deficient semiconductors, their LSPRs could be tuned to the NIR region.^22^ Integration of those two constituents into one nano-entity could combine the advantages of both components and improve their potential application performance.^23,24^

The plasmonic properties of hetero-nanostructures are strongly dependent on the size, shape, composition, arrangement, and distribution of each component. Hybrid nanomaterials with asymmetric architecture offer richer surface chemistry and a higher number of active sites, compared to symmetric core–shell structures, which permits their properties and functionality to be modulated to a higher extent.^23,24^ Besides the formation of a Schottky barrier for effective charge separation and plasmon–exciton interactions, joining of a noble metal and plasmonic semiconductor also results in plasmonic coupling between the core and shell materials, making these hybrids dual plasmonic nanomaterials. Such hetero-nanostructures showed enhanced NIR absorption compared to that of nonstoichiometric copper chalcogenides themselves due to the presence of the surface-enhanced near-field at the Au surface and its effect on the collective oscillation of free holes in the semiconductor.^25,26^

Combination of two distinctive plasmonic nanomaterials provides the ability to selectively control the LSPR of each constituent. The spectral position of the plasmon resonance response from metal NPs upon interaction with light can be altered by changing their geometry or effective dielectric environment.^27^ Isotropic metal NPs demonstrate a single dipolar LSPR, while nanoparticles with anisotropic shapes, such as rods, cubes, plates, triangles, and stars exhibit multiple higher-order LSPR modes. In non-spherical NPs, ununiform charge distribution gives rise to large electromagnetic field enhancements localized near the tips, corners, and edges.^28,29^ The plasmon resonance frequency of noble metal NPs is pre-set during synthesis and cannot be substantially changed after fabrication. On the other hand, the free carrier density in p-doped semiconductors can be controlled during fabrication and post-synthesis by exposure to oxidizing agents,^30^ cation intercalation and cation exchange,^31^ ligand exchange,^32^ and thermal treatment.^21^ Also, the wavelengths of LSPR mode in semiconductors can be adjusted using the same strategy as for noble metals, particularly by changing the size, or aspect ratio for anisotropic NPs.^33^ For binary copper chalcogenides, the extinction peak is moved farther into the IR region and its intensity decreases with approaching the Cu_2_E (E = S, Se, Te) stoichiometry.^22,34,35^

In our previously reported studies,^36^ we successfully demonstrated such plasmonic coupling effect between two dissimilar building blocks on the accelerated photocatalytic reactions in heterostructures using isotropic Au nanoparticles as the core and Cu_2−*x*_Se as the shell materials, respectively. Although the superior activities of dual plasmonic nanostructures in photocatalysis were ascribed to the enhanced electromagnetic field intensities as shown in our work, the limited tunability in the extent of plasmonic coupling due to the low spectral overlap of two distinctive plasmon resonances between the spherical Au core and the shell of Cu_2−*x*_Se compromised our further understanding of the role of the strengths of plasmonic coupling in photocatalysis. In such context, hetero-nanostructures with more efficient plasmonic coupling need to be designed and fabricated to allow us to elucidate the effect of such fundamental crosstalk on molecule-transformation processes. Utilization of plasmonic nanoparticles with anisotropic shapes and structures can ensure more efficient coupling since the LSPRs of the metallic component can be experimentally fine-tuned to the NIR range, where the LSPRs of vacancy-doped copper chalcogenides reside to maximize the band overlap in spectra. Thus, anisotropic noble metal–vacancy-doped semiconductor hetero-nanostructures become suitable objects for research of plasmonic effects, and fundamental understanding of different LSPR couplings, and their influence on characteristics of such nanomaterials. It must be noted that the aim is not to compare structures based on isotropic and anisotropic Au NPs. Instead, we studied the effect of the bands overlap extent in anisotropic structures by synthesizing Au NRs cores with different aspect ratios to obtain Au NRs-1@Cu_2−*x*_Se with partial band overlap and Au NRs-2@Cu_2−*x*_Se with full overlap.

In this work, we studied dual plasmon coupling in anisotropic Au@Cu_2−*x*_Se using Au NRs as the core and demonstrated its effect on photocatalytic activities. By experimentally tuning the aspect ratios, two monodisperse Au NRs (Au NRs-1 and Au NRs-2) with longitudinal plasmon resonance peaks centered at 680 nm and 880 nm, respectively were fabricated and used as the anisotropic cores to construct 2 sets of dual-plasmonic heteronanostructures (Au NRs-1@Cu_2−*x*_Se and Au NRs-2@Cu_2−*x*_Se) *via* unidirectional overgrowth of the Cu_2−*x*_Se shell on one-side of Au NRs in the presence of capping agents CTAC. Owing to the varying extents of spectral overlap between 2 distinctive LSPRs in such dual-plasmonic heteronanostructures, the effectiveness of plasmonic coupling was maneuvered in the current work. To better investigate the sole effect of plasmonic coupling on the kinetics of photocatalysis without complications, we deliberately tuned the shell thicknesses of Au NRs-1@Cu_2−*x*_Se and Au NRs-2@Cu_2−*x*_Se hybrids to have the same dimensions when comparing their rate constants obtained from the trajectories of photocatalytic reactions under illumination. The photocatalytic performance of hybrid nanostructures with the same shell thicknesses of 25 nm was firstly studied and then the outcomes were substantiated by the structures with thicker shells (66 nm). Based on experimental results, the best photocatalytic performance among studied Au NRs@Cu_2−*x*_Se nanostructures was demonstrated by nanomaterials with the strongest plasmonic coupling and the thickest shell due to more effective near-field enhancement, efficient interfacial charge transfer, and larger surface area.

To study the dependence of the coupling strength between two dissimilar LSPRs present in Au NRs@Cu_2−*x*_Se hetero-nanostructures on their properties, nanomaterials based on the anisotropic gold core, specifically Au NRs, and nonstoichiometric copper selenide with increasing shell thickness were designed. Uniform Au NRs with different dimensions were synthesized as shown in TEM micrographs (Fig. S1b and c, ESI†). Au NRs-1 with an aspect ratio of 2.4 had an average length of 85.3 ± 6.7 nm and a diameter of 36.3 ± 2.8 nm and Au NRs-2 with an aspect ratio of 3.8 had an average length of 58.5 ± 8.9 nm and a diameter of 15.3 ± 1.5 nm (Fig. S2, ESI†). Longitudinal and transverse LSPR bands of Au NRs-1 were positioned at 683 nm and 518 nm, respectively. Manipulating the dimensions of anisotropic plasmonic nanomaterials allowed us to further tune the resonance frequency. Thus, Au NRs-2 extinction peaks were centred at 887 nm and 507 nm in the spectrum for longitudinal and transverse LSPRs, respectively (Fig. S1a, ESI†).

To fabricate Au NRs@Cu_2−*x*_Se hetero-nanostructures, a simple Se-mediated approach in aqueous media with cetyltrimethylammonium chloride (CTAC) as the surfactant was used. CTAC serves not only as the stabilizer, but also determines the morphology of the produced nanostructures.^37^ First, an amorphous Se shell was deposited on the Au NRs core by reducing SeO_2_ with ascorbic acid (AA). Both extinction peaks of Au NRs underwent bathochromic shift, which was caused by the higher refractive index of Se than that of water (Fig. S3, ESI†). Corresponding color changes were observed in a series of colloidal solutions (Fig. S4, ESI†). The high affinity of Se to Au and its amorphous structure help to avoid synthetic issues related to gold and copper selenide lattice mismatch (Fig. S5, ESI†). After addition of the copper precursor in the presence of AA to previously formed Au NRs@Se NPs, the nonstoichiometric copper selenide crystalline shell was formed. This synthetic method permits us to vary the semiconductor shell thickness by varying the amount of Se-precursor used as well as simultaneously control of its doping level by timing the oxidation by air after addition of CuSO_4_.

In addition to the two resonance peaks of the Au NR core, in the extinction spectra of Au NRs@Cu_2−*x*_Se one more intense and relatively broad band in the NIR region from Cu_2−*x*_Se could be observed (Fig. 1). Au NRs@Cu_2−*x*_Se nanocomposites display stronger extinction in visible and NIR spectral regions than the individual Au and Cu_2−*x*_Se components. As the Cu_2−*x*_Se shell grows, the intensity of its LSPR band increased mainly due to the larger cross-section of the nanostructures. While bathochromic spectral movement of Au NR extinction bands was due to the combination of factors, which are the coupling between the plasmons in the Au core and the Cu_2−*x*_Se shell and larger refractive indices of the Cu_2−*x*_Se surrounding Au NR core compared to water,^38^ the *λ*_max_ position change of copper selenide was determined by the concentration of free holes in the shell. The stoichiometry of the semiconductor is determined by the time of oxidation in air and overall shell thickness. Because the same volume of SeO_2_ resulted in various shell sizes for Au NRs with different aspect ratios but the stirring time after CuSO_4_ addition was fixed based on corresponding volumes, different trends in the NIR peak position were observed. As can be concluded from the obtained spectra, the density of free charges could be easily tuned, so that longitudinal LSPRs of Au NRs and Cu_2−*x*_Se superimpose to a certain degree at NIR frequencies. As a result, there were only two LSPR peaks observed in the spectra of Au NR-2@Cu_2−*x*_Se colloids, which means that the longitudinal LSPR band of the Au NR core overlaps completely with the Cu_2−*x*_Se LSPR band (Fig. 1b).

**Fig. 1:**
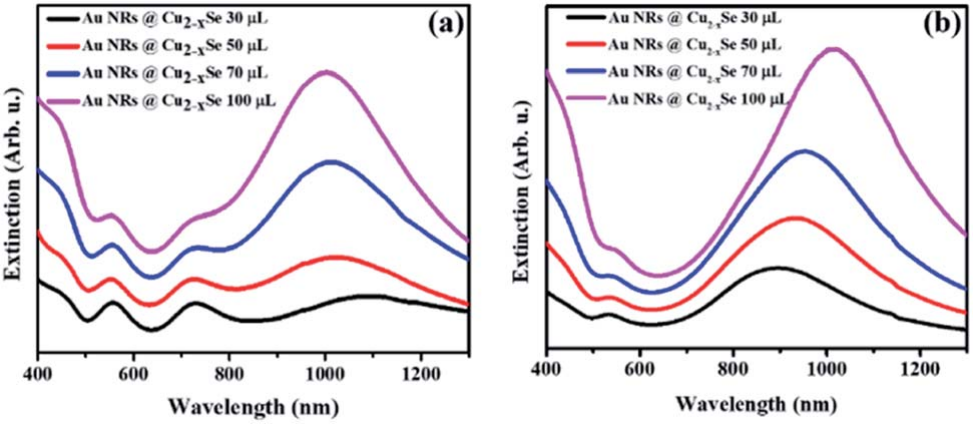
UV-Vis-NIR extinction spectra of aqueous solutions of Au NRs@Cu_2−*x*_Se nanoparticles with (a) Au NRs-1 and (b) Au NRs-2. Corresponding volume of 0.01 M SeO_2_ used for synthesis is noted in the figure.

Thus, through elaborate synthetic design of the Au NR core and Cu_2−*x*_Se shell the LSPRs of both can be accurately tuned to the same wavelength. Such maximum degree coupling of the LSPRs arising from collective oscillations of different charged species may enhance charge carrier transfer between metallic and semiconductor phases and therefore, positively affect the properties of the nanocomposites.

The semiconductor shell is attached to the core and mainly grows on one of the lateral sides of gold NRs, which was observed in obtained TEM images (Fig. 2). The Cu_2−*x*_Se domain had a pseudo-hemispherical shape with the highest thickness at the centre, decreasing, and curving to the ends. Due to the blocking effect of high energy Au NR surface facets by the Cl^−^ anion from CTAC and surface strains caused by Se overgrowth, the final core@shell products were asymmetric. The contrast difference of the dark Au nanorods and the gray Cu_2−*x*_Se in TEM micrographs confirms that Au NRs@Cu_2−*x*_Se nanocomposites possess two domains and are well dispersed. The dimensions of the Au NR core remained the same after Cu_2−*x*_Se shell deposition. The copper selenide shell exhibits good crystallinity. The lattice spacing with 0.33 nm was determined from well-defined fringes in the high-resolution TEM image and assigned to the (111) planes of copper selenide (Fig. S6, ESI†).

**Fig. 2:**
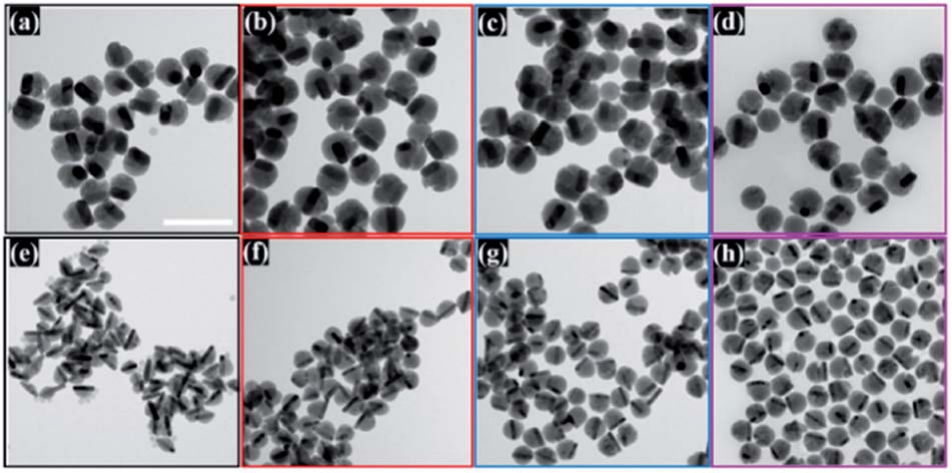
TEM images of Au NRs@Cu_2−*x*_Se nanostructures with (a)–(d) the Au NRs-1 core and increasing Cu_2−*x*_Se shell size obtained using (a) 30 μL, (b) 50 μL, (c) 70 μL, and (d) 100 μL of 0.01 M SeO_2_ and with (e)–(h) the Au NRs-2 core and increasing Cu_2−*x*_Se shell size obtained using (e) 30 μL, (f) 50 μL, (g) 70 μL, and (h) 100 μL of 0.01 M SeO_2_. Scale bar corresponds to 200 nm.

The Cu_2−*x*_Se shell thickness was adjusted by changing the SeO_2_ concentration and measured directly from the TEM images. To get the same thickness of the Cu_2−*x*_Se shell on Au NRs with different dimensions and concentrations in the colloid, the amount of Se precursor had to be changed. To obtain 25 nm-thick Cu_2−*x*_Se on Au NRs-1 only 10 μL of 10 mM SeO_2_ was used, compared to 30 μL of 10 mM SeO_2_ to form the semiconductor shell of the same thickness on Au NRs-2 (Fig. S7, ESI†). Among prepared hetero-nanostructures, two pairs of samples were selected for photocatalytic studies based on the same copper selenide domain size. Specifically, Au NR@Cu_2−*x*_Se hetero-nanostructures had shell thicknesses of ∼25 nm and ∼66 nm. Distribution histograms of the shell thickness indicate that the relative standard deviation is in the 4.8% and 7.2% range (Fig. S8, ESI†). Also, before conducting the photocatalytic experiment, the concentration of Au NRs@Cu_2−*x*_Se was adjusted based on ICP-OES results for gold content (Fig. S9, ESI†). Different aspect ratios of Au NRs, various concentrations of copper vacancies in the semiconductor, changing size of both core and shell contribute to strong NIR extinction and permit achieving different degrees of spectral band overlap in the nanosystems studied in the photocatalytic experiment (Fig. S10, ESI†). Au NRs@Cu_2−*x*_Se hybrid nanostructures showed improved photocatalytic efficiency by increasing the carrier separation and life-time due to the charge transfer process through the Schottky barrier between the metal and semiconductor and the plasmon-mediated enhancement effect. The anisotropic gold core with facets partially exposed to the reaction medium and the non-stoichiometric copper deficient shell produce charge transporters, undergo diverse surface chemistry, and generate reactive oxygen species (ROS). The photocatalytic performance of the Au NRs@Cu_2−*x*_Se nanostructures was evaluated by setting a model reaction of the photodegradation of RhB dye. The reactive species generated during the photocatalytic process, ˙O_2_^−^ and ˙OH, destroy different chemical bonds present in the RhB molecule in four steps: *N*-deethylation, cleavage of the C=N bond, ring-opening with possible isomerization, and mineralization.^39^ The temporal UV-Vis spectra of the degraded RhB solution under light illumination with wavelengths longer than 420 nm were recorded (Fig. S11, ESI†). The absorbance of RhB at a wavelength of 553 nm decreased during the reaction time, which indicates that RhB was gradually degraded. The process of photocatalytic degradation obeys the pseudo-first-order kinetic model
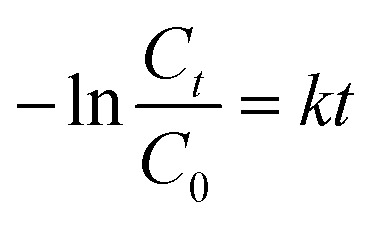
where *k* is the rate constant, *C*_0_ and *C*_*t*_ are the concentrations of RhB after reaching adsorption equilibrium in the dark after 1 h stirring and after irradiation time *t*, respectively (Fig. 3a). The rate constants for the Au NRs@Cu_2−*x*_Se samples with shell size around 25 nm were determined to be 1.52 × 10^−3^ min^−1^ and 2.28 × 10^−3^ min^−1^ for structures based on Au NRs-1 and Au NRs-2, respectively (Fig. 3b). For the Au NR@Cu_2−*x*_Se nanocatalysts with the thicker shell, the *k* values were 2.97 × 10^−3^ min^−1^ and 4.19 × 10^−3^ min^−1^ for samples based on Au NRs-1 and Au NRs-2, respectively (Fig. 3c).

**Fig. 3:**
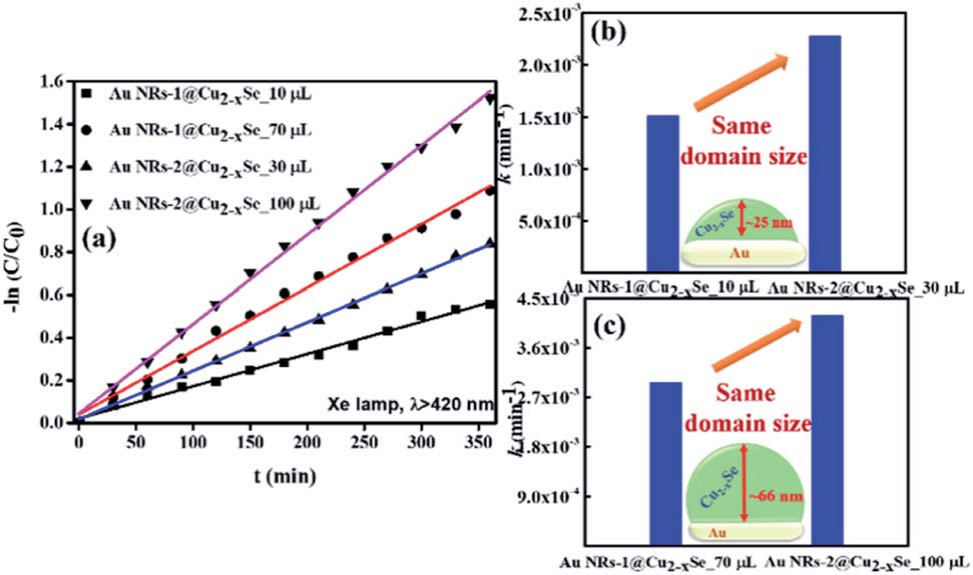
(a) First-order kinetics for RhB degradation under illumination (*λ* > 420 nm) using Au NRs@Cu_2−*x*_Se nanoparticles as catalysts. (b) and (c) The comparison of the rate constants (*k*) of photocatalytic degradation of RhB in the presence of Au NRs-1@Cu_2−*x*_Se and Au NRs-2@Cu_2−*x*_Se with shell thicknesses of (b) ∼25 nm and (c) ∼66 nm.

The data suggest that the photocatalytic activity is dependent on the shell thickness for metal–semiconductor hybrid nanostructures and the extent to which LSPR peak positions of both components coincide. The dark test with photocatalysts demonstrated that RhB could be degraded, but at a significantly lower rate (Fig. S12, ESI†). These results could be explained by the synergistic effects of surface area and plasmonic coupling between LSPRs of Au and Cu_2−*x*_Se under irradiation. The quantification of specific contributions of these effects is now underway in our lab.

In conclusion, we studied the plasmon coupling effect in Au NRs@Cu_2−*x*_Se nanosystems. For this, we fabricated dual plasmonic hetero-nanostructures with different optical properties. At first, Au NRs with two aspect ratios of 2.4 and 3.8 were obtained and used as a core for hetero-nucleation of nonstoichiometric copper selenide. Deposition of the semiconductor was done *via* a simple and reproducible selenium mediated method in water. We were able to control the thickness of the Cu_2−*x*_Se shell and examine its effect, together with the Au core's anisotropy and its dimensions, on the properties of the hybrid nanocrystals. This approach provides an adjustable and flexible protocol for systematically and predictably manipulating the heterostructure morphology, tuning the LSPRs bands' spectral positions, and ensuring a certain extent of their overlap. The dual-plasmon phenomenon has been observed experimentally in such hetero-nanostructures resulting in differences in extinction spectra of produced hybrids and its counterparts. By managing the strength of the coupling effect in Au NRs@Cu_2−*x*_Se nanosystems, we distinguished its influence on photocatalytic properties. Maximum spectral overlap of gold and copper selenide LPSRs ensures the strongest coupling of plasmons of different origin that come from two constituents, which leads to an enhanced local field, plasmon-induced hot-electron transfer under light illumination at the heterostructure interface, and advanced photocatalytic activities.

## Author contributions

H. J. conceived and designed the experiments. M. I. performed the synthesis and characterization of nanoparticles and analysed the data for the photocatalytic reactions. H. J. and M. I. drafted the manuscript. All authors proofread, commented on, and approved the final version of the manuscript.

## Conflicts of interest

The authors declare that they have no conflict of interest in this work.

## Supplementary Material

NA-004-D2NA00126H-s001
