# Supplementary material for: Anisotropic dual-plasmonic hetero-nanostructures with tunable plasmonic coupling effects
Source: Nanoscale Adv. 2022 Apr 12;4(12):2632–6. doi: 10.1039/d2na00126h (PMC9419501; doi:10.1039/d2na00126h)
Supplement: NA-004-D2NA00126H-s001 [file NA-004-D2NA00126H-s001.pdf]

Electronic Supplementary Material (ESI) for Nanoscale Advances.
This journal is © The Royal Society of Chemistry 2022

Anisotropic Dual-Plasmonic Hetero-Nanostructures with Tunable Plasmonic Coupling Effects

Mariia Ivanchenko[a] and Hao Jing*[a]

[a]*Department of Chemistry and Biochemistry, George Mason University, Fairfax, VA 22030, USA*

Chemicals and Materials

Hexadecyltrimethylammonium bromide (CTAB, > 98.0%), hexadecyltrimethylammonium chloride (CTAC, > 95.0%), sodium oleate (NaOL, > 97.0%), hydrogen tetrachloroaurate (III) trihydrate ($HAuCl_4 \cdot 3H_2O$, 99.99%), L-ascorbic acid (AA, ≥ 99.0%), silver nitrate ($AgNO_3$, >99.0 %), sodium borohydride ($NaBH_4$, 99.99%), hydrochloric acid (HCl, 37 wt. % in water, 12.1 M), selenium dioxide (≥ 99.9%), copper (II) sulfate (99.99%), Rhodamine B (RhB, ≥ 95 %). All the chemicals were used as received without further purification. The deionized water used in the experiment was ultra-pure (MilliQ, 18 MΩ).

Synthesis of Gold NRs

Seed solution preparation: 0.6 mL of freshly prepared 0.01 M $NaBH_4$ solution was rapidly injected in the mixture of 5 mL of 0.5 mM $HAuCl_4$ and 5 mL of 0.2 M hexadecyltrimethylammonium bromide (CTAB) aqueous solution under vigorous stirring. Immediate color change from yellow to light brown was observed. After 2 minutes stirring at 1200 rpm, seed solution was left undisturbed for 30 minutes.

Growth solution: 0.7 g of CTAB and 0.1234 g sodium oleate were dissolved in 25 mL of water at ~60 °C. After dissolution, the mixture cooled down to 30 °C and this temperature was maintained during entire synthesis. Then, 1.8 mL of 4 mM $AgNO_3$ solution was added, and the mixture was left undisturbed for 15 minutes. When the time was over, 25 mL of 1 mM $HAuCl_4$ solution was

added, while stirring at 700 rpm. The mixture was stirred for 90 minutes, and the disappearance of yellow color was observed. The pH value was adjusted by addition of 0.21 mL (Au NRs-1 with aspect ratio 2.4) or 0.3 mL (Au NRs-2 with aspect ratio 3.8) of concentrated HCl and stirring at 400 rpm for 15 minutes. Next, 0.125 mL of 0.064 M ascorbic acid was injected, while the mixture was vigorously stirred at 1200 rpm. After 30 s, a 0.08 mL of seed solution was introduced into the growth mixture, while maintaining vigorous stirring. The mixture was stirred for 30 s and left undisturbed at 30°C overnight. Obtained Au NRs were washed by centrifugation at 7000 rpm for 30 min. After the removal of the supernatant, product was redispersed in 10 mL of 0.1 M hexadecyltrimethylammonium chloride (CTAC).

Synthesis of Au NRs@$Cu_{2-x}Se$

Au NRs-1 with aspect ratio 2.4: 0.25 mL as prepared Au nanorods were diluted with 1.25 mL of water and mixed with 0.5 mL of 0.02 M CTAC and 0.2 mL of 0.1 M L-ascorbic acid (AA) aqueous solutions at 40 °C. While stirring at 1200 rpm, 10, 30, 50, 70, or 100 μL of 0.01 M SeO_2 aqueous solution was added dropwise utilizing a syringe pump with an injection rate of 10 μL/min. The reaction was allowed to proceed for 10 mins after the injection had been finished. Then, Au@Se dispersion was washed twice with water to remove any free Se NPs and redispersed in 2 mL of 5 mM CTAC. 15 μL of 0.2 M $CuSO_4$ and 0.2 mL of 0.1 M AA aqueous solutions were added into the colloid simultaneously at room temperature. The mixture was left under vigorous stirring at 1200 rpm for 40-60 mins (time increases for thicker Se shell). The resulting products were purified through centrifugation at 4000 rpm for 5 mins and redispersed in water.

Au NRs-2 with aspect ratio 3.8: followed the same protocol, except volumes of 0.01 M SeO_2 aqueous solution used were 30, 50, 70, 100 μL and centrifugation was performed at 7000 rpm for 5 mins.

Evaluation of the photocatalytic performance

The photocatalytic activity of the Au NRs@$Cu_{2-x}Se$ nanostructures was evaluated by the photodegradation of Rhodamine B (RhB) under a Xe lamp irradiation equipped with an ultraviolet-cut (λ > 420 nm) filter at 24 °C. Photocatalytic reactions were conducted in a cylindrical quartz reactor equipped with the outer shell for water circulation. Concentration of gold in colloidal solutions was adjusted to 214.14 mg/L based on results of ICP-OES analysis. 5 mmol/L solution of RhB was added to 0.25 mL of Au NRs@$Cu_{2-x}Se$ dispersion to get a total volume of 2 mL. Prior to illumination, the mixture was magnetically stirred in the dark for 1 h to allow for RhB molecules adsorption onto the catalyst surface. At irradiation time intervals of 30 min, dispersion was collected and centrifuged to remove the photocatalyst particles. After the catalyst was separated, the ultraviolet spectrum of the supernatant was recorded. Then, nanocatalysts were redispersed in the supernatant and illuminated again. During the photodegradation process, the RhB concentration was monitored by measuring the absorbance value at 553 nm.

Characterization and Instrumentation

Transmission electron microscopy (TEM and HRTEM) images were obtained on an FEI Titan 80–300 analytical transmission electron microscope and a JEOL JEM-1400Flash microscope operating at 120 kV. The TEM samples were prepared by dropping colloidal solutions of nanoparticles onto the surface of 300-mesh carbon-coated copper grids. The optical extinction spectra of colloidal nanoparticles were recorded at room temperature on a Shimadzu UV-2600 Plus spectrophotometer equipped with an integrating sphere in plastic cuvettes of 1-cm optical path length. ICP-OES analysis was performed utilizing SpectroBlue-FMT36 ICP OES spectrometer.

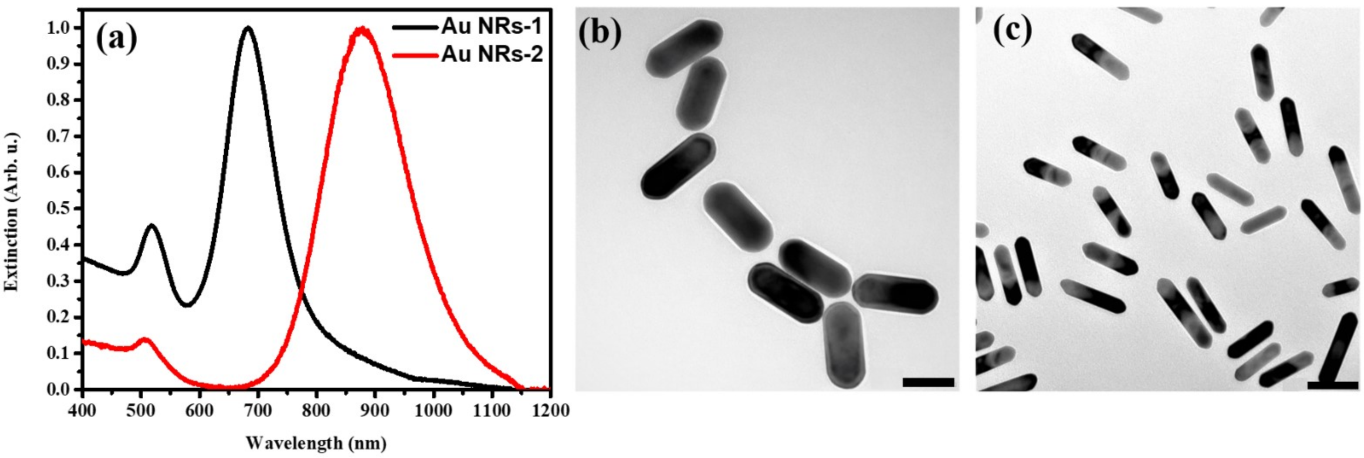

Fig. S1. (a) Normalized UV–vis–NIR extinction spectra of aqueous Au NRs colloids. TEM images of (b) Au NRs-1 and (c) Au NRs-2. Scale bars correspond to 50 nm.

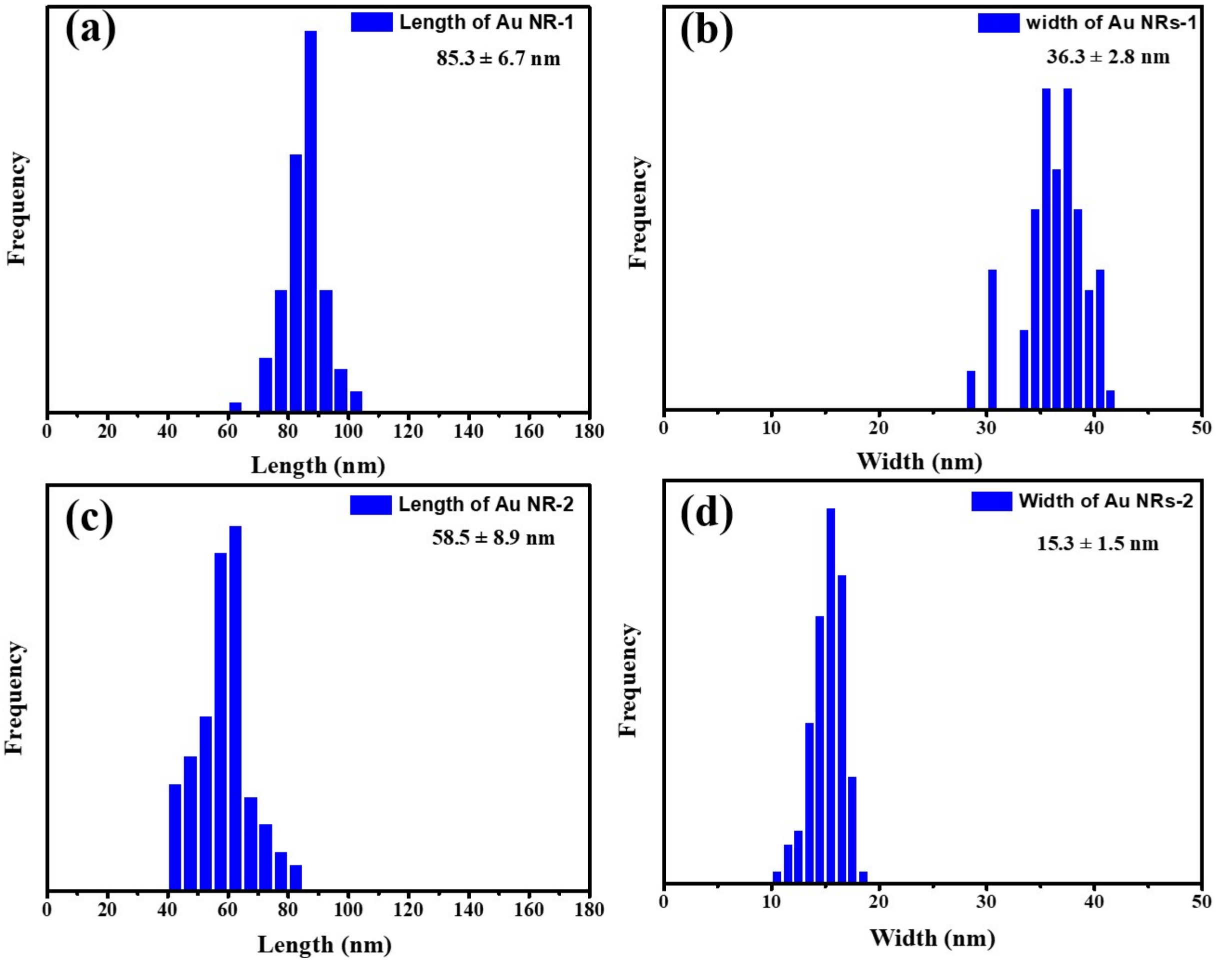

Fig. S2. Size distributions of Au NRs used as a core. (a) Length distribution of Au NR-1. (b) Width distribution of Au NR-1 (A.R. 2.4). (c) Length distribution of Au NR-2. (d) Width distribution of Au NR-2 (A.R. 3.8).

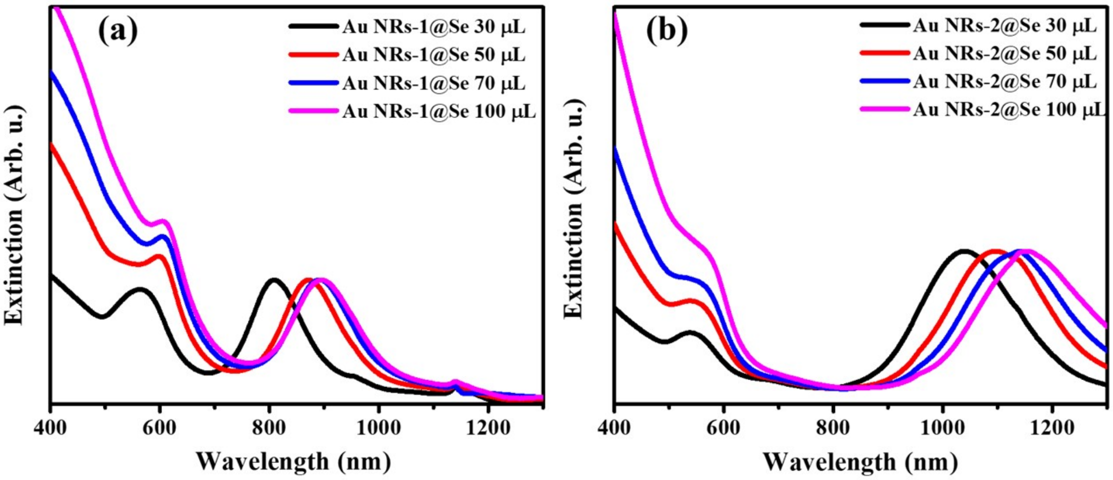

Fig. S3. Normalized UV–vis–NIR extinction spectra of aqueous solutions of intermediate Au NRs@Se nanoparticles with (a) Au NRs-1 (b) Au NRs-2. Corresponding volumes of 0.01 M SeO_2 used for synthesis are noted on the figure.

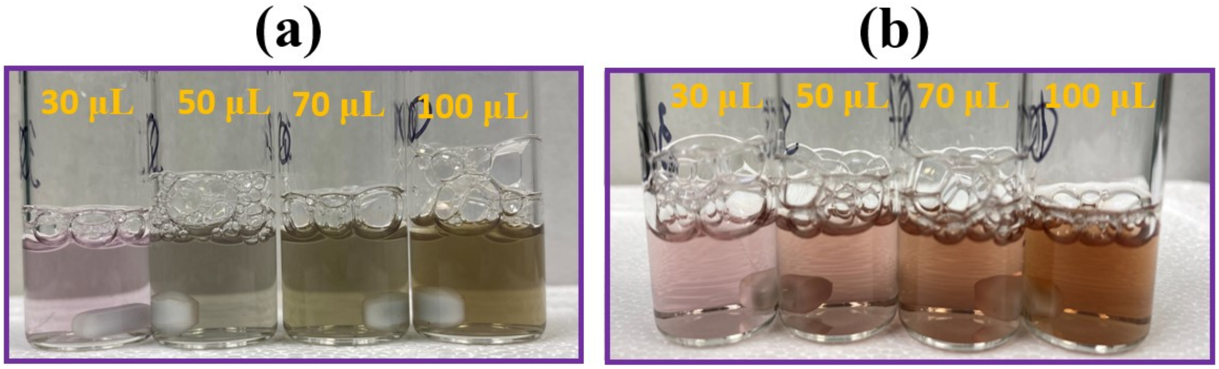

Fig. S4. Pictures of the color evolution of the intermediate Au NRs@Se nanoparticles with (a) Au NRs-1 (b) Au NRs-2. Corresponding volumes of 0.01 M SeO_2 used for synthesis are noted.

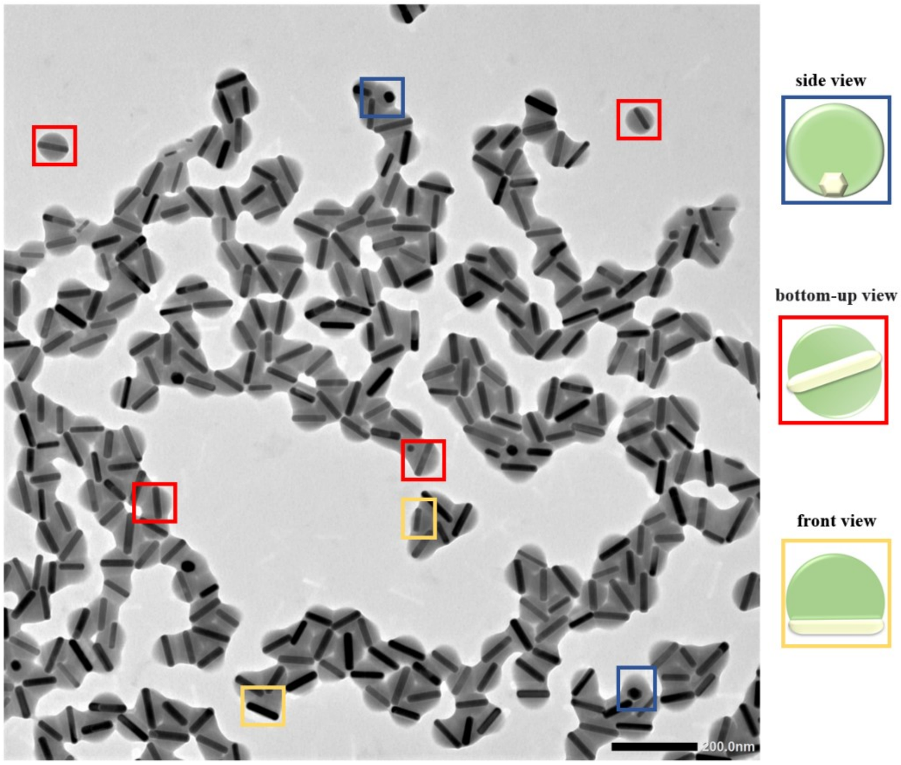

Fig. S5. TEM image of Au NRs-2@Se nanostructures obtained using 70 µL of 0.01 M SeO_2. Scale bar corresponds to 200 nm.

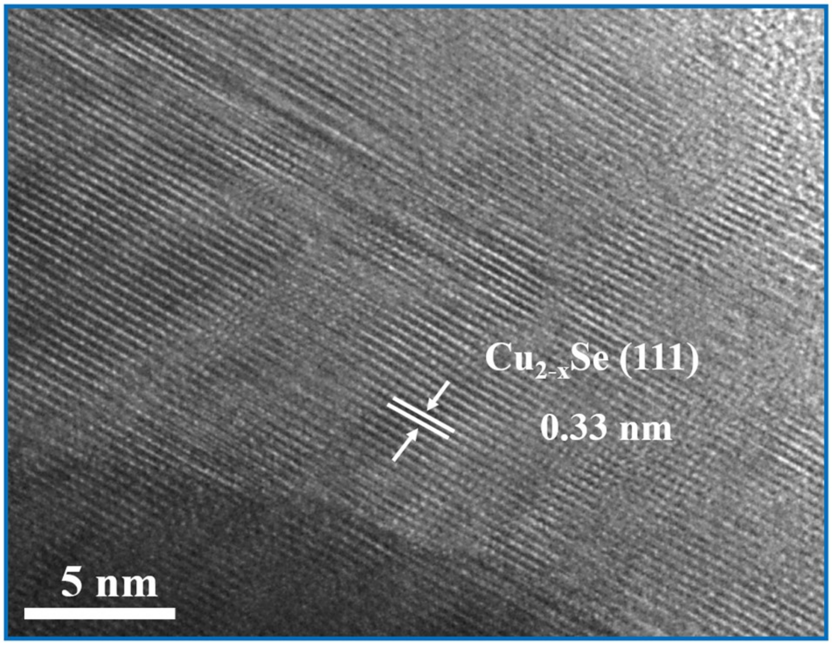

Fig. S6. High resolution TEM (HRTEM) image of Au NRs-1@Cu_{2-x}Se obtained using 70 μL 0.01M SeO_2 showing the interplanar distance of (111) planes of Cu_{2-x}Se determined to be 0.33 nm.

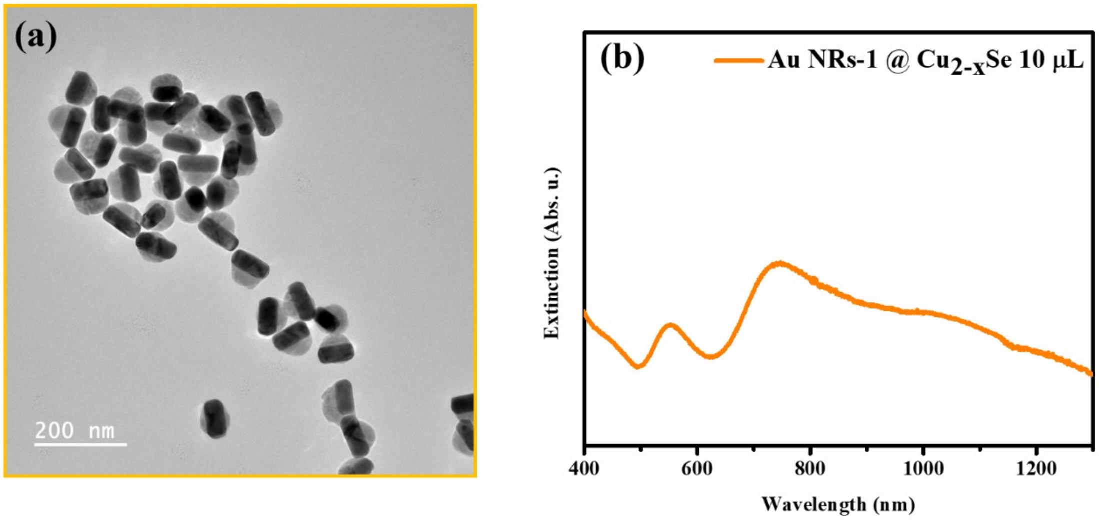

Fig. S7. (a) TEM micrographs of Au NRs-1@Cu_{2-x}Se-10 μL; (b) Extinction spectrum of Au NRs@Cu_{2-x}Se nanoparticles synthesized using 10 μL of 0.01 M SeO_2.

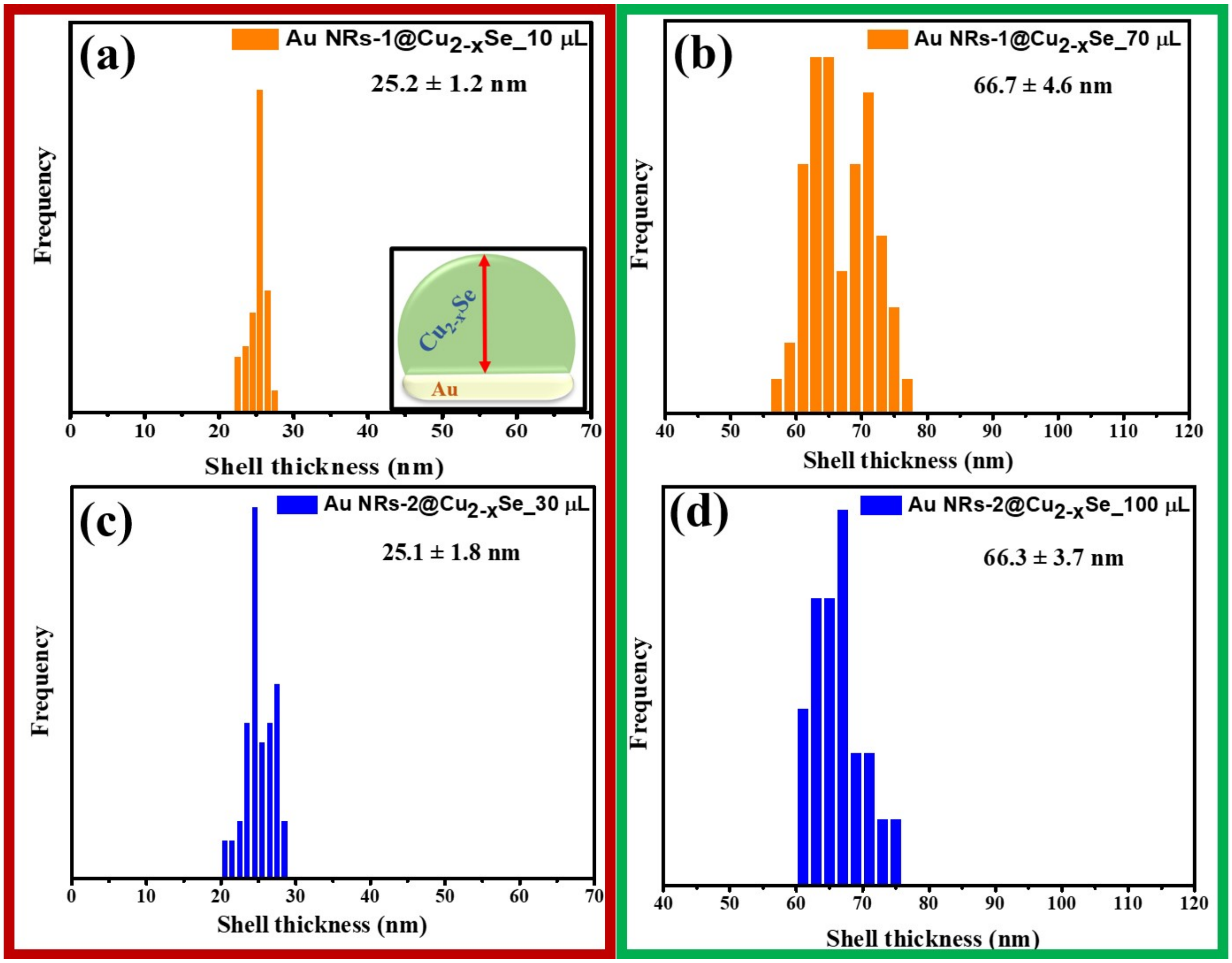

Fig. S8. Distributions of $Cu_{2-x}Se$ shell thickness obtained from TEM images of Au NRs@$Cu_{2-x}Se$ NPs used as photocatalysts. (a) Au NRs-1@$Cu_{2-x}Se$ synthesized using 10 μL of 0.01 M SeO_2. Insert: demonstration of the measuring approach. (b) Au NRs-1@$Cu_{2-x}Se$ synthesized using 70 μL of 0.01 M SeO_2. (c) Au NRs-2@$Cu_{2-x}Se$ synthesized using 30 μL of 0.01 M SeO_2. (d) Au NRs-2@$Cu_{2-x}Se$ synthesized using 100 μL of 0.01 M SeO_2.

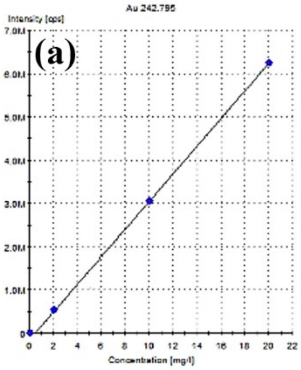

(b)

Samples	Concentrations based on ICP-OES results
**Au NRs-1**	**107.07 mg/L**
**Au NRs-2**	**127.35 mg/L**

Fig. S9. (a) Calibration curve for ICP-OES measurements of Au-content. (b) Concentration of Au in colloids obtained from ICP-OES analysis.

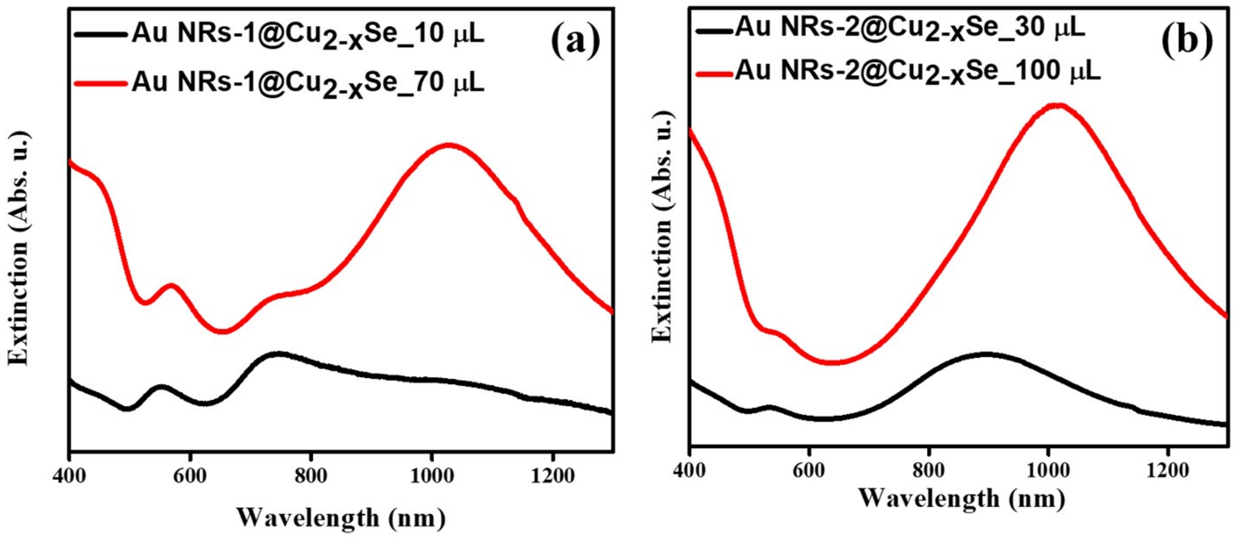

Fig. S10. Extinction spectrum of (a) Au NRs-1@$Cu_{2-x}Se$ NPs and (b) Au NRs-2@$Cu_{2-x}Se$ NPs with different shell thickness demonstrating the difference is in plasmonic coupling strengths between Au NRs and $Cu_{2-x}Se$ domain.

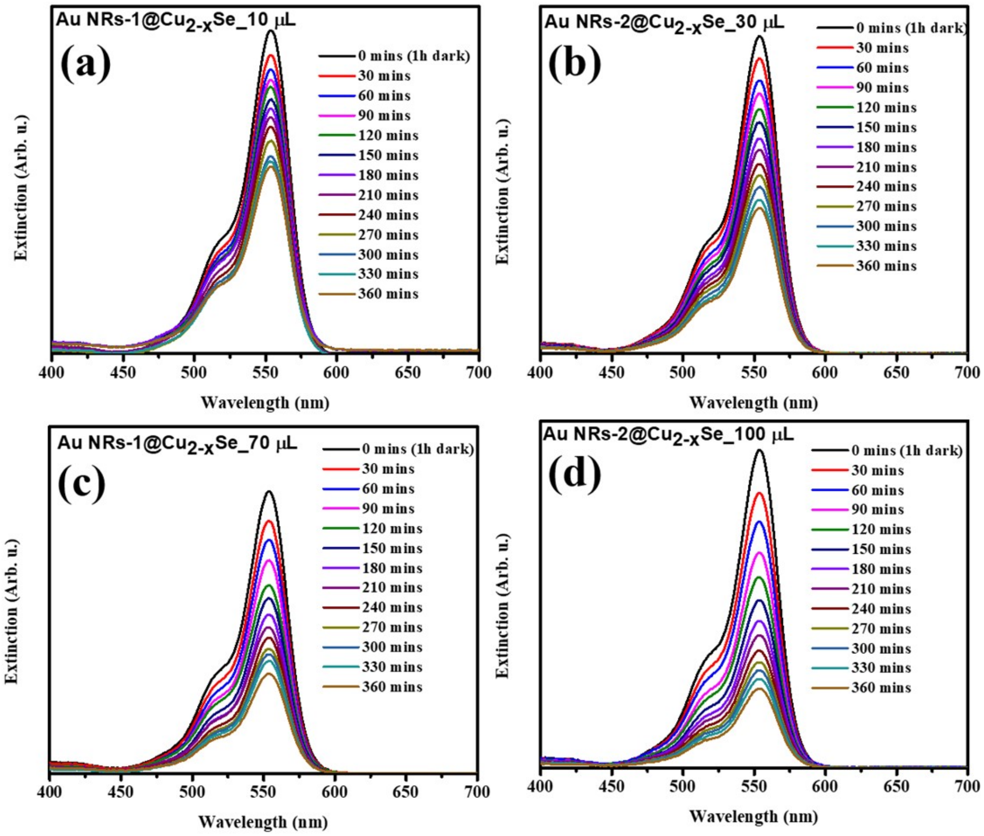

Fig. S11. Time resolved absorption spectra of photocatalytic degradation of RhB molecules in the presence of (a) Au NRs-1@$Cu_{2-x}Se$ synthesized using 10 μL of 0.01 M SeO_2 (b) Au NRs-2@$Cu_{2-x}Se$ synthesized using 30 μL of 0.01 M SeO_2 (c) Au NRs-1@$Cu_{2-x}Se$ synthesized using 70 μL of 0.01 M SeO_2 (d) Au NRs-2@$Cu_{2-x}Se$ synthesized using 100 μL of 0.01 M SeO_2 when optical long-pass filter with cut-off wavelength of 420 nm was used during the illumination.

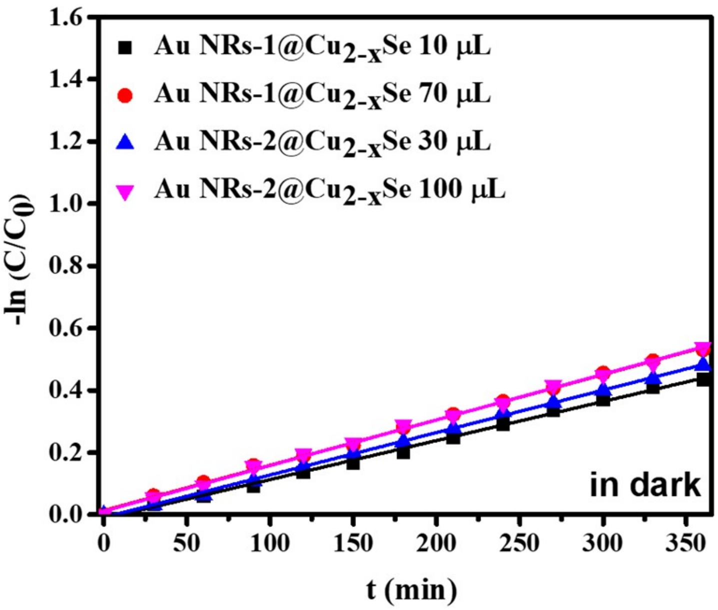

Fig. S12. First-order linear transformations, $–\ln(C/C_0)$, for RhB degradation reactions using of AuNRs@$Cu_{2−x}Se$ NPs as catalysts in the dark.
